# Systemic Therapy for Patients with HER2-Positive Breast Cancer and Brain Metastases: A Systematic Review and Meta-Analysis

**DOI:** 10.3390/cancers14225612

**Published:** 2022-11-15

**Authors:** Inge M. Werter, Sharon Remmelzwaal, George L. Burchell, Tanja D. de Gruijl, Inge R. Konings, Hans J. van der Vliet, C. Willemien Menke-van der Houven van Oordt

**Affiliations:** 1 Department of Medical Oncology/Internal Medicine, Rijnstate Hospital, Wagnerlaan 55, 6815 AD Arnhem, The Netherlands; 2 Department of Epidemiology & Data Science, Amsterdam UMC, Location VUmc, De Boelelaan 1117, 1081 HV Amsterdam, The Netherlands; 3 Medical Library, Amsterdam UMC, Location VUmc, De Boelelaan 1117, 1081 HV Amsterdam, The Netherlands; 4 Department of Medical Oncology, Cancer Center Amsterdam, Amsterdam UMC, Location VUmc, De Boelelaan 1117, 1081 HV Amsterdam, The Netherlands; 5 Lava Therapeutics, Yalelaan 60, 3584 CM Utrecht, The Netherlands

**Keywords:** breast cancer, brain metastases, HER2, lapatinib, trastuzumab-deruxtecan, tucatinib, pyrotinib, trastuzumab-emtansine, neratinib

## Abstract

**Simple Summary:**

Patients with HER2-positive metastatic breast cancer develop brain metastases in up to 30% of cases. The aim of this systematic review and meta-analysis was to determine the effect of different systemic therapies in patients with HER2-positive metastatic breast cancer and brain metastases, acknowledging the heterogeneity and sometimes low quality of 51 included studies. Tucatinib (combined with trastuzumab and capecitabine) and trastuzumab-deruxtecan appear to constitute the most effective systemic therapy, while pyrotinib might be an option in Asian patients. Preferably, future research will comprise of randomized controlled trials, including patients with active and/or inactive brain metastases.

**Abstract:**

Aim: Patients with HER2-positive (HER2+) metastatic breast cancer (mBC) develop brain metastases (BM) in up to 30% of cases. Treatment of patients with BM can consist of local treatment (surgery and/or radiotherapy) and/or systemic treatment. We undertook a systematic review and meta-analysis to determine the effect of different systemic therapies in patients with HER2+ mBC and BM. Methods: A systematic search was performed in the databases PubMed, Embase.com, Clarivate Analytics/Web of Science Core Collection and the Wiley/Cochrane Library. Eligible articles included prospective or retrospective studies reporting on the effect of systemic therapy on objective response rate (ORR) and/or median progression free survival (mPFS) in patients with HER2+ mBC and BM. The timeframe within the databases was from inception to 19 January 2022. Fixed-effects meta-analyses were used. Quality appraisal was performed using the ROBINS-I tool. Results: Fifty-one studies were included, involving 3118 patients. Most studies, which contained the largest patient numbers, but also often carried a moderate-serious risk of bias, investigated lapatinib and capecitabine (LC), trastuzumab-emtansine (T-DM1) or pyrotinib. The best quality data and/or highest ORR were described with tucatinib (combined with trastuzumab and capecitabine, TTC) and trastuzumab-deruxtecan (T-DXd). TTC demonstrated an ORR of 47.3% in patients with asymptomatic and/or active BM. T-DXd achieved a pooled ORR of 64% (95% CI 43–85%, I^2^ 0%) in a heavily pretreated population with asymptomatic BM (3 studies, *n* = 96). Conclusions: Though our meta-analysis should be interpreted with caution due to the heterogeneity of included studies and a related serious risk of bias, this review provides a comprehensive overview of all currently available systemic treatment options. T-Dxd and TTC that appear to constitute the most effective systemic therapy in patients with HER2+ mBC and BM, while pyrotinib might be an option in Asian patients.

## 1. Introduction

Metastatic breast cancer (mBC) is highly prevalent, 20% of mBC patients have HER2-positive (HER2+) mBC [1], 30% of which develop brain metastases (BM) [2]. This results in an incidence of BM in HER2+ mBC per patient-year of 13% [2]. Over the years, the survival of patients with HER2+ mBC and baseline BM improved significantly, from a median survival of 3–6 months to almost 30–38 months [3,4,5,6]. Patients who received anti-HER2 treatment had longer median OS than those without [7]. However, patients with BM still have a worse median survival compared to patients without BM [8]. Due to the blood-brain barrier (BBB) and the blood-tumor barrier (BTB), development of systemic treatments that are effective in patients with BM has been challenging, as large molecule biologic drugs supposedly have a limited ability to cross the (intact) BBB. The BBB is the term used to describe the unique characteristics of the endothelial cells of blood vessels that vascularize the central nervous system (CNS), which tightly regulates the movement of ions, molecules, and cells between the blood vasculature and the parenchyma, which is critical for neuronal function and protection [9]. The BTB describes the modifications to the BBB in patients with BM and primary brain tumors [9].

The cornerstone of the treatment of BM consists of local treatment modalities like surgery and/or stereotactic radiotherapy, often combined with systemic treatment. Besides a direct cytotoxic effect, systemic treatments can also exert a radio-sensitizing effect [10,11,12,13]. Systemic therapies for patients with HER2+ mBC include chemotherapy (e.g., taxanes), monoclonal antibodies (mAbs; eg. trastuzumab and pertuzumab (TP)), antibody-drug conjugates (ADCs; e.g., trastuzumab-emtansine (T-DM1), trastuzumab-deruxtecan (T-DXd)) and small molecule tyrosine kinase inhibitors (TKIs; e.g., Lapatinib, Pyrotinib, Neratinib, Afatinib, Cabozantinib and Tucatinib). Given the number of available therapies for patients with HER2+ mBC and the high prevalence of BM in these patients, it is important to understand which treatment is the most effective in terms of response rate and/or survival. In addition to intracranial objective response rates (ORR), intracranial efficacy of a systemic treatment can also be deducted from its capacity to successfully postpone or prevent the development of BM.

The combination of TP and a taxane was investigated in the Cleopatra trial and demonstrated to be an effective first line therapy prolonging survival in HER2+ mBC [14,15]. Trastuzumab is a humanized mAb specific for extracellular domain IV of HER2. Pertuzumab is a humanized mAb specific for extracellular domain II of HER2, and thereby blocks a binding pocket necessary for receptor dimerization with HER3 [16]. While trastuzumab was considered not to cross the BBB due to its high molecular weight, it does appear to have intracranial efficacy, as it has been implicated to slow down the development of BM, and the use of trastuzumab is associated with a longer survival in mBC patients with BM [5,17]. Indeed, a study using ^89^Zr-trastuzumab confirms that trastuzumab can access BM, possibly due to a compromised BBB [18]. Other imaging studies using ^89^Zr-pertuzumab demonstrated that pertuzumab can also access BM, and similarly, ^11^C lapatinib has also been shown to cross the BBB [19,20].

Since most patients with HER2+ mBC do not initially present with BM, they will probably have been treated with trastuzumab-based regimens before BM manifested. Currently used HER2 directed therapy in case of BM are mostly based on expert opinion, as patients with BM, especially symptomatic BM, were frequently excluded from trials. Though there have been earlier reviews on this subject [21,22,23,24], including one meta-analysis that focused on the combination of lapatinib and capecitabine (LC) in patients with BM of HER2+ mBC [25], our study, to the best of our knowledge, is the most complete overview comprising all different systemic therapies available to patients with HER2+ mBC and (a)symptomatic BM. Despite the high risk of bias and heterogeneity in the current meta-analysis, the data presented will support clinical decision making for these patients.

## 2. Methods

### 2.1. Search Strategy and Selection Criteria

This systematic review and meta-analysis was performed in accordance with the Preferred Reporting Items for Systematic Reviews and Meta-Analyses (PRISMA) guidelines. A systematic search was performed in the databases PubMed, Embase.com, Clarivate Analytics/Web of Science Core Collection and the Wiley/Cochrane Library. The timeframe within the databases was from inception to 19th January 2022 and conducted by GB and IW. Eligible articles included prospective or retrospective studies reporting on the effect of systemic therapies on ORR and/or median progression free (mPFS) in patients with HER2+ mBC and BM. Studies were grouped based on investigational treatment arm, irrespective of active or inactive BM, treatment line, study design or quality. The search included keywords and free text terms for synonyms of ‘breast neoplasm’ combined with synonyms of ‘HER2′ combined with synonyms of ‘brain metastases’. Reviews, animal studies, comments, letters, editorials, qualitative studies, case reports and case series (of less than 10 patients) were excluded from the search. A full overview of the search terms per database can be found in the supplementary information (see Tables S1–S4). No limitations on date or language were applied in the search. Selection of studies was done by two reviewers independently (IW and HV) based on title and/or abstract. Disagreement between reviewers was resolved by a third reviewer (WM).

### 2.2. Data Analysis

Data was extracted from published reports. Besides ORR and mPFS, data about intervention, line of therapy, previous local treatment, extra CNS disease, amount of BM and mOS was extracted if available, no assumptions were made in case of missing data. Meta-analysis was performed when a minimum of three studies reported similar effect measures for similar outcomes and similar interventions. Specifically, for the meta-analyses on mPFS and median overall survival (mOS), we needed months of survival and the respective confidence intervals. For the meta-analyses on ORR, we needed numbers of response and total numbers of the groups. Summary estimates were computed by either using random-effects meta-analysis for the months of survival, or fixed-effects meta-analysis with Clopper-Pearson derived confidence intervals and Freeman-Tukey double arcsine transformation to stabilize inter-study variance for the ORR. Heterogeneity between studies was assessed by using the I^2^ statistic, where we considered an I^2^ value greater than 50% indicative of substantial heterogeneity. Subgroup analyses were not performed, due to low volume of studies. We performed sensitivity analyses if abstract-only articles were available, due to low quality of most included studies, we were not able to perform sensitivity analyses based on quality. When a meta-analysis was not possible because of a low number of studies, we used a descriptive synthesis. All analyses and plots were performed in RStudio version 4.0.3. using the ‘meta’ package [26].

We used the ROBINS-I tool to assess the quality of the included studies (non-randomized studies and RCTs) [27]. Additionally, we used domain 1 of the Risk of Bias 2 (RoB 2) tool (risk of bias arising from the randomization process) for the included RCTs [28]. This assessment was done at study level and performed by two independent reviewers (IW and WM). Disagreement between reviewers was resolved by a third reviewer (HV). Risk-of-bias plots were created using the robvis-tool [29].

## 3. Results

**Figure 1:**
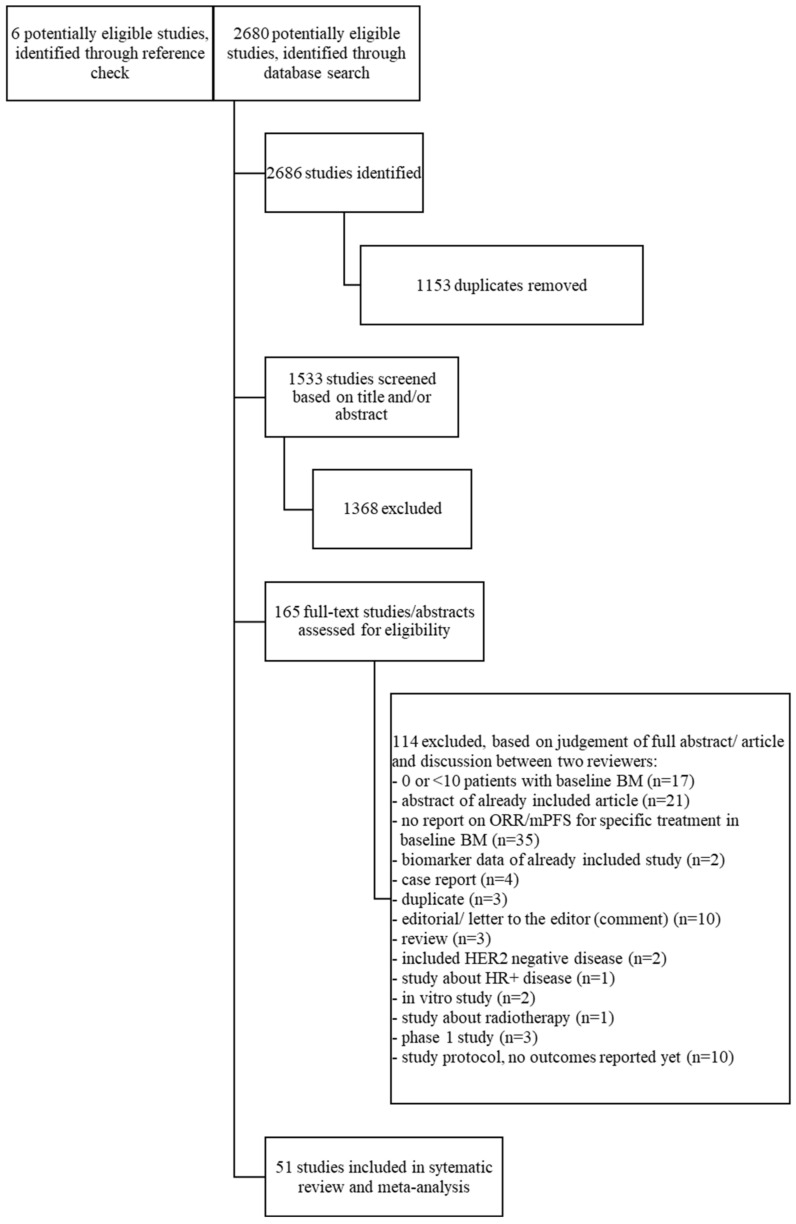
Flow chart of the search strategy.

A flow diagram for the search strategy is shown in Figure 1. The search yielded 2686 studies, after deduplication, 1533 studies were identified, of which 1368 were excluded based on title and/or abstract. Reasons for exclusion were type of study (reviews, preclinical studies, phase 1 studies and studies comprising <10 patients) or the subject of the study (no HER2+ mBC, no patients with baseline BM, outcome not specifically related to type of systemic treatment and studies on biomarkers and genes and studies investigating local treatments). The 165 studies were discussed more thoroughly by the two reviewers, leading to 51 relevant articles involving 3118 patients included in the systematic review. Characteristics of the included studies are shown in Table 1 (BEEP, afatinib, neratinib, everolimus, cabozantinib, tucatinib, T-Dxd and trastuzumab/pertuzumab), Table 2 (T-DM1), Table 3 (lapatinib) and Table 4 (pyrotinib).

**Figure 2:**
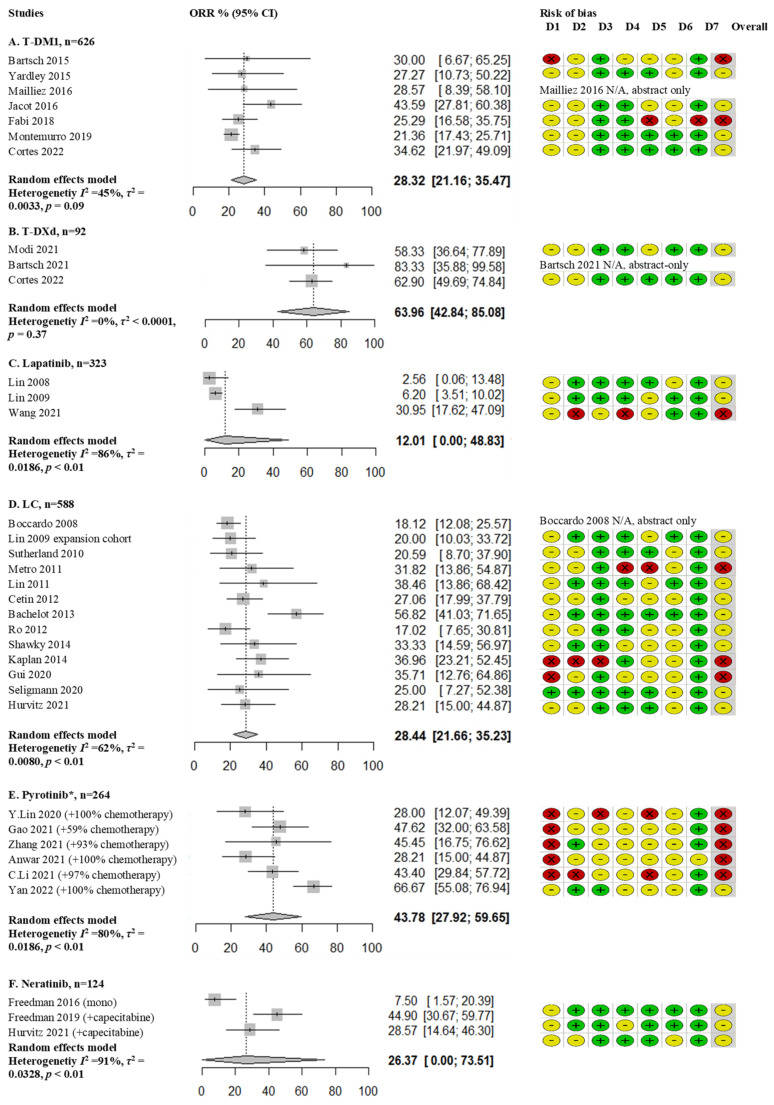
Pooled ORR meta-analysis per drug (combination) and quality assessment of risk of bias (**A**) Trastuzumab-emtansine (T-DM1); (**B**) Trastuzumab-deruxtecan (T-DXd); (**C**) Lapatinib; (**D**) Lapatinib + capecitabine (LC); (**E**) Pyrotinib; (**F**) Neratinib; * amount of patients receiving combination therapy with chemotherapy, mostly capecitabine (see Table 4).

**Figure 3:**
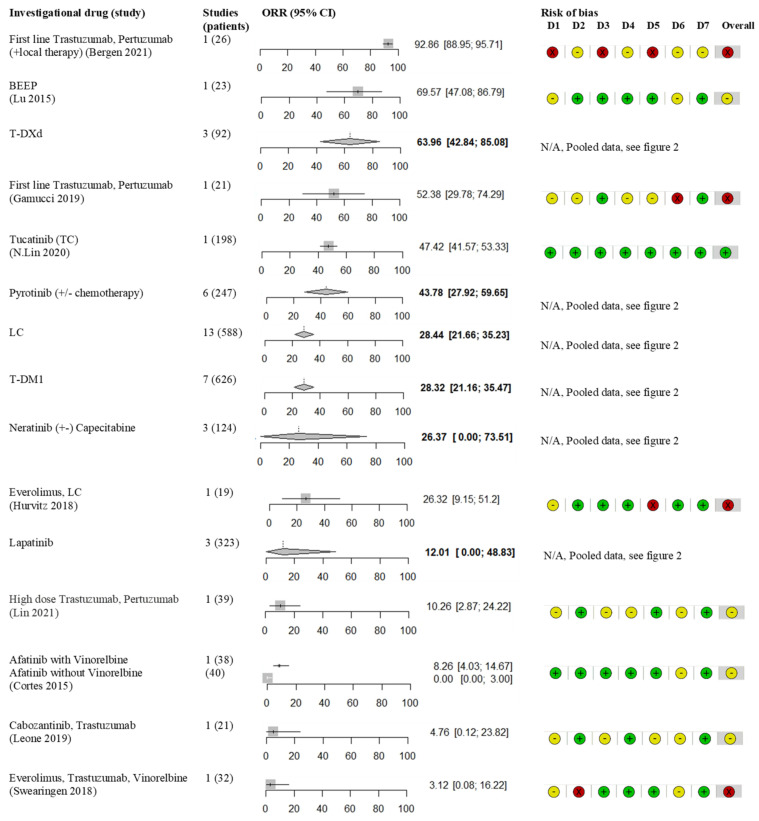
ORR for all drug (combinations). Overview of single studies, pooled meta-analysis and quality assessment of risk of bias.

Of the 51 included studies, 4 studies were abstract-only studies. Consequently, there was not enough information for risk of bias interpretation. The other 47 articles comprised of 8 retrospective analysis of randomized studies; namely, 3 open label randomized phase 2 studies (Lux Breast3, Lantern and EGF107671), 3 open label randomized phase 3 studies (Emilia, NALA and Destiny Breast 03) and 2 double blind randomized phase 3 studies (Phoenix and HER2CLIMB). In addition, one open-label phase-3b single arm study was included (Kamilla). Further studies consisted of 14 single arm phase 2 studies, 1 case series, 4 open-label extended access program studies and 23 retrospective observational single arm studies. Risk of bias was assessed for all included studies (Figure 2 and Figure 3). A common cause of bias for many included studies resulted from the different criteria used for assessing progression of BM, and often this outcome was not a primary or secondary endpoint. T-DM1 and LC studies were mostly of moderate-serious risk of bias (Figure 2B,D). The pyrotinib studies were all of serious risk of bias, except for the Phoenix trial (Figure 2E). Especially the HER2CLIMB trial had a low risk of bias (Figure 3). Despite presenting a complete overview of all treatment options to date, the reader should realize that due to different trial designs (prospective, retrospective, randomized and non-randomized), different treatment lines and inclusion of both active and inactive BM, the presented meta-analysis was hampered by bias and heterogeneity.

Domains:

D1: Bias due to confounding

D2: Bias due to selection of participants

D3: Bias in classification of interventions

D4: Bias due to deviations from intended interventions

D5: Bias due to missing data

D6: Bias in measurements of outcomes

D7: Bias in selection of reported results


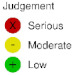


Domains:

D1: Bias due to confounding

D2: Bias due to selection of participants

D3: Bias in classification of interventions

D4: Bias due to deviations from intended interventions

D5: Bias due to missing data

D6: Bias in measurements of outcomes

D7: Bias in selection of reported results


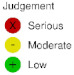


### 3.1. Monoclonal Antibodies

Two studies, investigating 47 patients, assessed the efficacy of first line TP and a taxane (Table 1). In the first line setting, local treatment of BM is standard of care, so these results should be interpreted for the combination. In the subset of 21 patients with baseline (inactive) BM in the retrospective Reper study, an ORR of 52.4% was achieved (Figure 3) and a mPFS of 20 months (95% CI 13–27 months) [44]. The retrospective study by Bergen et al. [43] investigated the effect of different first-line systemic treatments for 252 patients with HER2+ mBC and BM. Of all included patients, 26 patients received first line TP combined with local therapy with or without chemotherapy, leading to an ORR of 92.9% (Figure 3), mPFS of 8.0 months (range 1–55 months) and mOS of 44 months (range 2–61 months). Both the Reper study as well as the study by Bergen et al. had a serious risk of bias (Figure 3) due to the retrospective design, no routine MRI scans of the brain and concomitant local therapies.

The single arm phase 2 PATRICIA study reported on high dose trastuzumab (HDT) (6 mg/kg weekly) in combination with pertuzumab after progression on standard dose trastuzumab and a median of three lines of previous therapy (*n* = 39) (Table 1) [42]. This was based on a preclinical mammary tumor graft model of HER2+ mBC, in which up to three times the regular dose of trastuzumab was needed to achieve similar responses in brain tumor grafts [79]. HDT was demonstrated to be safe but resulted in a low ORR of 11% (Figure 3).

### 3.2. Antibody Drug Conjugates

ADCs approved for the treatment of patients with HER2+ mBC are T-DM1 and T-DXd. T-DM1 contains the microtubule-inhibitory agent DM1 (derivative of maytansine) conjungated to trastuzumab [80]. T-DXd has the DNA topoisomerase I inhibitor deruxtecan conjugated to trastuzumab [81]. Compared to T-DM1, T-DXd has a higher antibody to drug ratio (8 versus (vs.) 3–4) and is probably more potent than T-DM1 as a result of the properties of its payload that facilitates penetration of deruxtecan through the cell membrane of the HER2+ tumor cells or neighboring cells, without requiring high HER2 expression levels [22,81].

**Figure 4:**
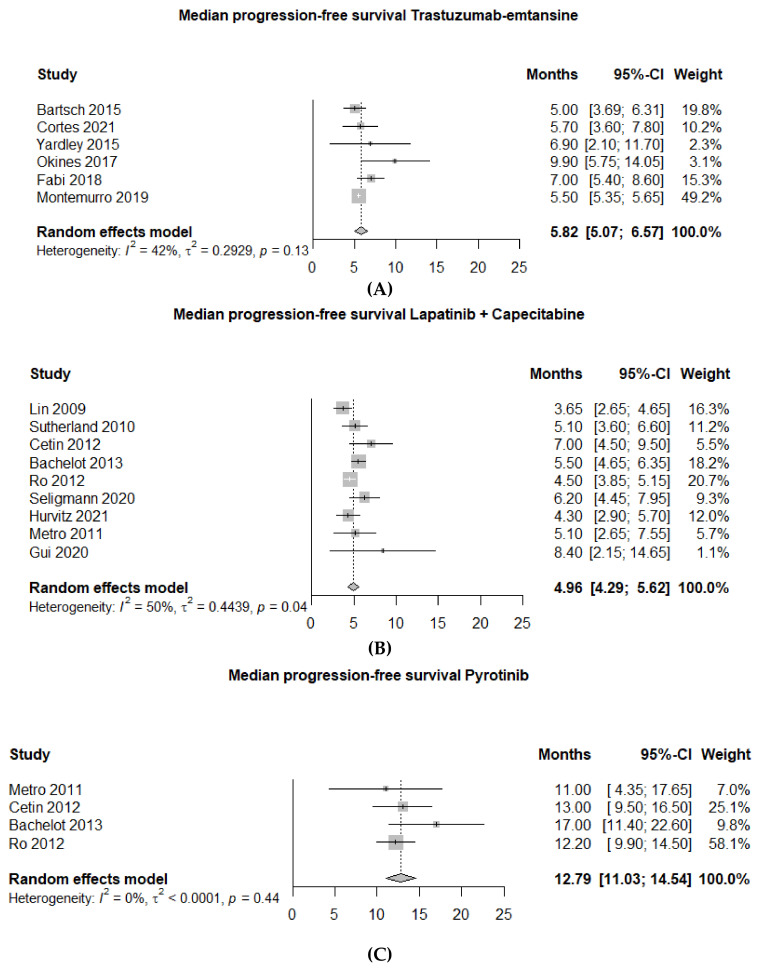
Pooled mPFS (months) meta-analysis; (**A**) Trastuzumab-emtansine (T-DM1); (**B**) Lapatinib + capecitabine (LC); (**C**) Pyrotinib.

T-DM1 was studied in 10 trials comprising 774 patients, mostly second line treatment (Table 2); 5 retrospective studies [48,49,50,51,53], 2 posthoc analyses of open label randomized phase 3 trials [41,45], 1 case series [46], 1 expanded access program [47] and 1 posthoc analysis of an open label single arm study [52] Pooled ORR was 28% (95% CI 21–35%; I^2^ 45%) and remained the same after excluding abstract-only articles in the sensitivity analysis (Figure 2A and Figure S1A). The Kamilla study demonstrated modest activity with an ORR of 21%. In this study, 6% of patients had an Eastern Cooperative Oncology Group (ECOG) performance status (PS) of 2, and a relatively low number of patients received prior pertuzumab (4%) or local treatment for BM (47%) [52]. mPFS was similar in all studies with a pooled mPFS of 5.8 months (95% CI 5.1–6.6 months; I^2^ 42%) (Figure 4A). mOS was reported in seven studies with a median of 15.3 months (range 8.5–26.8 months) (Table 2).

T-DXd was studied in 3 trials and 96 patients (Table 1); 2 single arm phase 2 trials [39,40] and a sub-analysis of an open label randomized phase 3 trial [41]. These studies included heavily pretreated patients with BM (54% pretreatment with HER2 targeting TKIs, TP and taxanes). The pooled ORR of the three studies was 64% (95% CI 43–85; I^2^ 0%) (Figure 2B). Most patients had stable BM. Efficacy in patients with BM was not an endpoint of the phase 2 DESTINY-Breast01 and phase 3 DESTINY-Breast03 studies. The single arm phase two TUXEDO-1 trial included patients with active BM and is still actively recruiting patients; data of the first 10 patients showed a promising ORR of 83.3%. The phase 2 DESTINY-Breast01 and phase 3 DESTINY-Breast03 studies reported on mPFS, ranging 15.0–18.1 months for patients with asymptomatic BM. There were no reports on mOS.

### 3.3. Tyrosine Kinase Inhibitors

Several TKIs have been evaluated in patients with HER2+ mBC. These TKIs differ in molecular weight, selectivity and reversibility of binding to HER2-protein, efficacy and their safety profile. Lapatinib is a reversible dual inhibitor of HER1/EGFR and HER2 [82]. Pyrotinib, neratinib and afatinib are all irreversible inhibitors of HER1/EGFR, HER2 and HER4 [31,83,84]. Cabozantinib is a multi-TKI inhibiting MET, VEGFR2, RET and other TKIs [37]. Tucatinib is a reversible and highly selective HER2 inhibitor [85].

There are two phase 2 studies [54,55] and one retrospective study [56], comprising 323 patients addressing lapatinib monotherapy. These three studies led to a pooled ORR of 12% (95% CI 0–49%; I^2^ 86%) and mPFS of 3.0 months (range 2.4–6.3 months) (Figure 2C and Table 3). The retrospective study by Wang et al. included patients in Chinese centers and demonstrated a relatively high ORR of 31% and mPFS of 6.3 months, independent of the line of therapy [56]. Of note, 26% of patients in this study received lapatinib combined with trastuzumab, and 59% of patients had not been previously treated with HER2 directed therapies. Moreover, only 59% had been treated with local therapy for BM compared to 95% in both studies by Lin et al. 2008 and 2009 [54,55]. The combination of lapatinib and trastuzumab was studied in the retrospective Trastyvere study, patients with BM had 3.8 months of mPFS and 15.2 months of mOS [57].

A total number of 16 studies, including 693 patients combined, which investigated LC have been included in the meta-analysis; 2 randomized phase 2 studies [61,69], 2 single arm phase 2 studies [63,66], 1 expansion cohort of a single arm phase 2 study [55], 3 expanded access program studies [58,59,64], 6 retrospective studies [60,62,65,67,68,70] and 2 posthoc analyses of open label phase 3 trials (Table 3) [34,45]. This demonstrated a pooled ORR of 28% (95% CI 21–35%; I^2^ 62%). After excluding abstract-only articles, the pooled ORR remained the same (Figure 2D and Figure S1B). Of note, though the Landscape trial demonstrated a high ORR of 57% [63], a high percentage of 78% of patients in this study were treated with LC in first or second line and all included patients had previously untreated BM. Survival analysis resulted in a pooled mPFS of 5.0 months (95% CI 4.3–5.6 months; I^2^ 50%) (Figure 4B) and a pooled mOS of 12.8 months (95% CI 11.0–14.5 months; I2 0%) (Figure S2).

In this meta-analysis, 9 studies investigating pyrotinib in a total of 321 Asian patients were included (Table 4); 1 double blind phase 3 study [71], 1 single arm phase 2 trial [78] and 7 retrospective studies [70,72,73,74,75,76,77]. Pooled ORR was 43% (95% CI 27–59%; I^2^ 80%) (Figure 2E). Most studies were of serious risk of bias due to retrospective design. Pyrotinib was mostly combined with capecitabine, but it was also given as monotherapy or in combination with other regimens. These studies were predominantly in second line, after trastuzumab-based therapy, patients had not received prior treatment with TP or T-DM1. Most studies did not report on previous local treatment for BM, and if reported, it was quite low in three studies (0%, 43%, 55%) (Table 4). Importantly, the phase 2 study by Yan et al. underscored the effect of prior radiotherapy for BM on ORR (radiotherapy naive cohort ORR of 74.6% vs. progressive disease after radiotherapy cohort ORR of 42.1%). Three studies were available for a pooled analysis of mPFS, which was 10.1 months (95% CI 4.3–15.8 months; I^2^ 88%) (Figure 4C). A mOS of 13.9 months was reported in one study; for the other studies, this information was lacking [75].

Neratinib was investigated as monotherapy in one phase 2 study (*n* = 40) [32] and in combination with capecitabine in two studies; a phase 2 study [33] and a posthoc analysis of a phase 3 trial [34] with a total of 100 patients (Table 1). Combining these three studies led to a heterogeneous meta-analysis due to difference in mono or combined intervention arms. In the neratinib monotherapy study, an ORR of 8% was demonstrated, while the two studies combining neratinib and capecitabine (NC) found an ORR of 29% and 49% (calculated from both lapatinib-naïve and lapatinib-treated cohort). Combining these three studies, a pooled ORR of 26% (95% CI 0–74%) was calculated (Figure 2F). For neratinib monotherapy, mPFS was 1.9 months vs. 5.5 and 5.6 months for NC. mOS was 8.7 months in the neratinib monotherapy study vs. 13.3 and 13.9 months for NC.

Afatinib was studied in one randomized phase 2 study as monotherapy (*n* = 40) and combined with vinorelbine (*n* = 38) [31]. Notably, in this study, only 41% of patients with BM also had extracranial disease (Table 1). Afatinib, alone or in combination, showed low efficacy with an ORR of 0% vs. 8% respectively (Figure 3) and a mPFS of 2.7 vs. 2.8 months, respectively. Due to low efficacy (and frequent adverse events), no further development of afatinib for HER2+ mBC is currently planned [31].

The combination of cabozantinib and trastuzumab was studied in one study with 21 heavily pretreated patients (Table 1) [37]. The investigators hypothesized that simultaneous targeting of both MET and VEGFR2 by cabozantinib might combine antivascular and anti-tumor activity. The ORR was 5% (Figure 3), mPFS 4.1 months (95% CI 2.8–6.2) and mOS 13.8 months (95% CI 8.2-NR). Cabozantinib therefore had insufficient activity and its use in this setting has not been further explored.

The combination of tucatinib, trastuzumab and capecitabine (TTC) was studied In 612 patients in the HER2CLIMB study [38]. A secondary endpoint of this double-blind randomized phase 3 trial was the efficacy of TTC in patients with (active and inactive) BM. Of the 612 patients, 291 patients had BM at baseline; 198 patients were treated with TTC, while 92 patients were treated with placebo, trastuzumab and capecitabine (Table 1, Figure 3). The ORR for TTC was 47.3% vs. 20.0% for placebo (*p* = 0.03). CNS mPFS for TTC was 9.9 vs. 4.2 months for placebo (HR 0.32; 95% CI 0.22–0.48; *p* < 0.0001) [85]. mOS for TTC was 18.1 vs. 12.0 months for placebo (HR 0.58; 95% CI 0.40–0.85; *p* = 0.005). Interestingly, 30 patients who had isolated CNS progression were allowed to continue systemic treatment according to the study protocol, after receiving local CNS therapy. In these patients, the median time from randomization to second disease progression or death was for TTC 15.9 vs. 9.7 months for placebo (HR 0.33; 95% CI 0.11–0.02).

### 3.4. Other Treatments

The combination of bevacizumab, etoposide and cisplatin (BEEP) was studied in 1 study of 23 patients (54.3% with an ECOG PS of 2 or 3), all of whom had progressive disease after prior whole brain radiotherapy (WBRT) (Table 1) [30]. It was the only study in this meta-analysis in which treatment did not consist of a HER2 targeting agent. The hypothesis was that a window period between bevacizumab and cytotoxic agents might enhance drug delivery to tumor tissue through bevacizumab-induced vascular normalization in patients with mBC and BM [30]. Patients in this study achieved an ORR of 69.6% (Figure 3), mPFS of 7.7 months (95% CI 6.6–8.8) and mOS of 11.8 months (95% CI 7.0–16.6). However, there is a serious risk of bias in outcome measurement due to the use of volumetric response criteria instead of RECIST or RANO, while part of the volumetric response might be due to effective treatment of radionecrosis by bevacizumab instead of representing effective anticancer treatment. Moreover, the study was constrained to the use of contrast-enhanced images for efficacy assessment instead of MRI T2/FLAIRE images because of post-WBRT diffuse white matter changes.

The effect of everolimus, a mTOR inhibitor was investigated in two studies, combinedly including 51 patients (Table 4). Previous results showed that hyperactivation of the PI3K/mTOR pathway during treatment with trastuzumab correlated with poor OS and increased risk of BM [86]. Thus, inhibition of the PI3K/mTOR pathway, combined with HER2-directed therapy, may yield more sustained responses for patients with HER2+ mBC and BM. Swearingen et al. combined everolimus with vinorelbine in 32 patients (97% prior local treatment for BM) and demonstrated an ORR of 4% (Figure 3), a mPFS of 3.9 months (95% CI 2.3–5.0) and a mOS of 12.1 months (95% CI 6.8–12.4); this schedule was deemed ineffective [35]. Hurvitz et al. combined everolimus with LC and included 19 patients (63% prior local treatment for BM) with less extracranial disease compared to the Swearingen study (42% vs. 66%); they reported an ORR of 28% (Figure 3), a mPFS of 6.2 months and a mOS of 24.2 months [36]. Accrual goals were not met. Importantly, 73% of patients were not pretreated with LC, thus the ORR of 28% could represent the ORR of LC instead of an additive effect of everolimus.

## 4. Discussion

We present a complete overview of systemic treatment options in HER2+ mBC with BM. Interpretation of the meta-analysis is limited by the high level of heterogeneity and risk of bias of the available studies. Best quality data and/or highest ORR in ≥2nd line were demonstrated in studies evaluating T-Dxd and tucatinib. We should take into account that patients in T-DM1, pyrotinib and LC studies received fewer prior treatments compared to T-Dxd and tucatinib. Concomitant local therapy, comedication, active/stable BM and ECOG status differed. Comparisons are mostly made based on CNS ORR, but not only BM response influences prognosis. The systemic disease status is also relevant and quite different in patients included in the different studies.

Based on the CLEOPATRA study data, the combination of TP and a taxane is considered standard first line therapy. In the CLEOPATRA study, median time to CNS PFS was delayed (15 vs. 11.9 months; HR 058; *p* = 0.0049). However, its efficacy for patients with baseline BM was only described in combination with local therapy in the RePer study and by Bergen et al. [43,44]. In later lines of therapy, reintroducing trastuzumab at a higher dosage was not effective.

Of all systemic therapies, T-DXd showed the highest pooled ORR (64%) in patients with HER2+ mBC and stable BM in ≥2nd line. Importantly, this effect was shown in a heavily pretreated population. Ongoing prospective studies on T-DXd will provide us with more data on its effect in patients with stable BM in the DESTINY Breast12 trial (ClinicalTrials.gov identifier: NCT04739761) and with active BM in the TUXEDO-1 trial (ClinicalTrials.gov identifier: NCT04752059), which reported promising first results [40]. In ≥2 line, TTC achieved a high ORR of 47% in the HER2CLIMB study; importantly this is the only well performed double blind randomized trial, demonstrating a mOS benefit for patients with BM. TTC is the only therapy studied for the treatment of active BM. At this moment, no comparative data between T-DXd and tucatinib are available. A direct comparison using currently available data is difficult, as in the HER2CLIMB trial no previous treatment with TKIs was allowed, in contrast to the Destiny Breast03 trial. The combination of tucatinib and T-DXd is currently being studied in the HER2CLIMB-4 study (ClinicalTrials.gov identifier: NCT04539938).

For Asian patients, pyrotinib is another ≥2nd line treatment option, demonstrating a pooled ORR of 43% and pooled mPFS of 10 months. However, most studies used retrospectively acquired data. Patients had received only a median of 1 prior treatment line, and almost no prior T-DM1 or pertuzumab. Moreover, ORR was mainly high in the radiotherapy-naïve group. Pyrotinib was directly compared to LC and capecitabine monotherapy in the Phoebe and Phenix trials, respectively. In these trials, pyrotinib demonstrated a superior efficacy in Asian patients in general. [71,84]. However, the number of patients with baseline BM in the Phoebe and Phenix trials was small, and occurrence of BM was comparable between pyrotinib and control arm (2%) [84], so more prospective data are needed regarding the efficacy of pyrotinib in patients with BM.

When opting for T-DM1 or LC in ≥3rd line, a comparable pooled ORR of 28% was found, although pooled mPFS was slightly longer for T-DM1 than for LC. Based on a direct comparison of T-DM1 and LC in the randomized phase 3 Emilia study, T-DM1 outperformed LC in terms of mOS in the selected group with baseline BM (26.8 vs. 12.9 months, *p* = 0.008) [45,80]. T-DM1 treated patients without baseline BM had a higher occurrence of BM over the course of their disease vs. LC treated patients (3.8% vs. 0.2%; *p* = NS). Regarding LC, the CEREBEL trial compared LC to trastuzumab and capecitabine (TC) in 501 patients with HER2+ mBC, and LC demonstrated a lower incidence of BM as first site of relapse than TC (3% vs. 5%; *p* = NS) [87].

NC demonstrated an ORR of 29% [34] and 49% [33], in two studies. When opting for treatment with either NC or LC, the randomized phase 3 NALA study can provide guidance, as it directly compared both therapies in 101 patients with HER2+ mBC and BM. Only a non-significant moderately improved mPFS was demonstrated for NC over LC and a comparable ORR was found [34]. However, patients treated with NC required significantly less interventions for BM (22.8% for NC vs. 29.2% for LC, *p* = 0.043), providing a hint of improved intracranial efficacy for NC over LC [88]. For NC, both cost and drug availability might be an issue as well as adverse events, as NC leads to diarrhea more frequently than LC [88].

Studies investigating everolimus, lapatinib monotherapy, cabozantinib or afatinib did not demonstrate a clinically relevant effect and/or included a low number of patients and should therefore currently not be considered for treating patients with HER2+ mBC with BM.

An important observation is that although the BBB is known to reduce efficacy of systemic treatments especially in preclinical models, with current TKIs and ADCs, we now have evidence of effective intracranial treatments for patients with BM, although mOS remains shorter than in patients without BM. Choices in sequential therapies can be made weighing ORR, mPFS, mOS, adverse events, availability and cost. Although the best order is not known, T-DXd and TTC are the most effective systemic treatment options to date in patients with HER2+ mBC and BM. In clinical practice, we would currently recommend T-DXd or TTC for second line treatment, realizing that both may become available for first line therapy in the near future. In case these drugs are not available, we would suggest pyrotinib for Asian patients. No further recommendations can be made due to low patient numbers and heterogeneity of the included studies.

This review provides an overview and insight in interpreting the efficacy of drugs in patients with HER2+ mBC and BM, acknowledging the heterogeneity and sometimes low quality of included studies. Preferably, future research will comprise of randomized controlled trials, including patients with active and/or inactive BM. Based on current knowledge, we would hypothesize that the most effective first line treatments in the future will consist of ADC’s. Importantly, in the current treatment landscape, patients receiving multiple lines of anti-HER2 therapy, administered after BM diagnosis, have a significantly improved mOS [89].

## Figures and Tables

**Table 1:** Characteristics of included studies on BEEP, afatinib, neratinib, everolimus, cabozantinib, tucatinib, T-Dxd and trastuzumab/pertuzumab (*N* = 15).

Study	Phase	Patients with Baseline BM (*n*)	Intervention (*n*)	Control (*n*)	Line of Therapy	Previous Local Treatment for BM (%)	Extra CNS Disease (%)	Number of BM	mPFS (Months)	mOS (Months)	CNS ORR %
Lu 2015 [30]	phase 2	23	BEEP (Bevacizumab, Etoposide, Cisplatin)	Single arm	median 3 (Range 1–8)	100%	94.3%		7.7 (95% CI 6.6–8.8)	11.8 (95% CI 7.0–16.6)	69.6%
Cortes 2015-lux breast 3 [31]	phase 2, randomised	38	Afatinib + Vinorelbine (38)	Investigator choice (43) or Afatinib (40)	1–2.31%; 3–4.68%	83%	41%	59% > 3	2.8	8.6	8.0%
Freedman 2016 [32]	phase 2	40	Neratinib	Single arm	0–2.17%; 3–4.83%	100%			1.9	8.7	8.0%
Freedman 2019 [33]	phase 2	49 (2 cohorts combined)	Neratinib + Capecitabine	Single arm	0.22%; 1.45%; ≥2 33%	92%	78%		5.5 (Range 0.8–18.8)	13.3 (Range 2.2–27.6)	44.9% 18 of 37.4 of 12
Hurvitz 2021-NALA [34]	phase 3b (posthoc)	51	Neratinib + Capecitabine (51)	Lapatinib + Capecitabine (50)	2.68%; ≥3.32%	80%	84%		5.6 (95% CI 3.7–7.1)	13.9 (95% CI 8.9–17.5)	28.6%
Swearingen 2018 [35]	phase 2	32	Everolimus + Trastuzumab + Vinorelbine	Single arm	median 2 (Range 0–7)	97%	66%		3.9 (95% CI 2.3–5.0)	12.1 (95% CI 6.8–12.4)	4.0%
Hurvitz 2018 [36]	phase 2	19	Everolimus + Lapatinib + Capecitabine	Single arm	median 2.5 (Range 0–11)	63%	42%		6.2	24.2	28.0%
Leone 2020 [37]	phase 2	21	Cabozantinib + Trastuzumab	Single arm	median 3 (Range 1–7)	81%	>48%		4.1 (95% CI 2.8–6.2)	13.8 (95% CI 8.2–NR)	5.0%
N.Lin 2020-HER2Climb [38]	phase 3	198	Tucatinib + Trastuzumab + Capecitabine (198)	Trastuzumab + Capecitabine (93)	median 3 (Range 1–14)	87%	97%		9.9 (95% CI 8.0–13.9)	18.1 (95% CI, 15.5–NR)	47.3%
Modi 2021 DESTINY-Breast01 [39]	phase 2	24	Fam-Trastuzumab deruxtecan	Single arm	median > 6			median 5	18.1 (95% CI 6.7–18.1)	NR	58.3%
Bartsch 2021-Tuxedo 1 [40]	phase 2	10	Fam-Trastuzumab deruxtecan	Single arm	70% > 2	60%					83.3%
Cortes 2022-Destiny breast-03 [41]	phase 3	62	Fam-Trastuzumab deruxtecan (62)	Trastuzumab-emtansine (52)	2.50%; 3 22%; >5.8%				15.0 (95% CI 12.6–22.2)		62.9%
Lin 2021-PATRICIA [42]	phase 2	39	High dose Trastuzumab/Pertuzumab (+28% Other )	Single arm	median 3 (Range 2–5)						11.0%
Bergen 2021 [43]	retrospective	26	Trastuzumab/Pertuzumab (60% + Chemo/Local Therapy)	Single arm	median 1 (Range 1–6)		80%		8.0 (Range 1.0–55.0)	44.0 (range 2.0–61.0)	92.9%
Gamucci 2019- RePer [44]	retrospective	21	Trastuzumab/Pertuzumab+ taxane	Single arm	Median 1	48%			20 (95% CI 13–27)		52.4%

**Table 2:** Characteristics of included Trastuzumab-emtansine (T-DM1) studies (n = 10).

Study	Phase	Patients with Baseline BM (*n*)	Intervention (*n*)	Control (*n*)	Line of Therapy	Previous Local Treatment for BM (%)	Extra CNS Disease (%)	Numberof BM	mPFS (Months)	mOS (Months)	CNS ORR %
Krop 2015-Emilia [45]	phase 3b (posthoc)	45	Trastuzumab-emtansine (45)	Lapatinib + Capecitabine (50)	median 3 (Range 1–13)	70%	79%		5.9	26.8	
Bartsch 2015 [46]	case series	10	Trastuzumab-emtansine	Single arm	1.40%; 2.60%	80%	90%	50% > 3	5.0 (95% CI 3.7–6.3)	8.5	30.0%
Yardley 2015 [47]	open label, prospective	26	Trastuzumab-emtansine	Single arm	median 8 (Range 3–23)				6.9 (95% CI 2.7–12.3)		27.3%
Mailliez 2016 [48]	retrospectief	14	Trastuzumab-emtansine	Single arm	median 2 (Range 0–7)				2.4 (Range 2.0–9.4)	9.1 (Range 3.7–24.8)	28.6%
Jacot 2016 [49]	retrospectief	39	Trastuzumab-emtansine	Single arm	median 2 (Range 0–8)	95%	82%	median 2 (Range 1–11)	6.1 (Range 5.2–18.3)	NR	43.6%
Okines 2018 [50]	retrospectief	16	Trastuzumab-emtansine	Single arm	median 2 (Range 0–6)	100%			9.9 (95% CI 3.9–12.2)	15.3 (95% CI 4.7–NR)	
Fabi 2018 [51]	retrospectief	87	Trastuzumab-emtansine	Single arm	1–2.51%; 3–4.49%	100%		25% > 3	7.0 (95% CI 5.4–8.6)	14.0 (95% CI 12.2–15.8)	25.3%
Montemurro 2019- Kamilla [52]	phase 3b (posthoc)	398	Trastuzumab-emtansine	Single arm	0–2.48%; 3–4.31%; ≥5.19%	47%	79%		5.5 (95% CI 5.3–5.6)	18.9 (95% CI 17.1–21.3)	21.4%
Bahceci 2021 [53]	retrospectief	87	Trastuzumab-emtansine	Single arm					9.0	19	
Cortes 2022-Destiny breast-03 [41]	phase 3b (posthoc)	52	Trastuzumab-emtansine (52)	Fam-Trastuzumab deruxtecan (62)	2				5.7 (95% CI 2.9–7.1)		34.0%

**Table 3:** Characteristics of included Lapatinib and/or Capecitabine studies (n = 20).

Study	Phase	Patients with Baseline BM (*n*)	Intervention (*n*)	Control (*n*)	Line of Therapy	Previous Local Treatment for BM (%)	Extra CNS Disease (%)	Number of BM	mPFS (Months)	mOS (Months)	CNS ORR %
Lin 2008 [54]	phase 2	39	Lapatinib	Single arm	1–2.25%; ≥3.75%	95%	>62%		3.0 (95% CI 2.3–3.7)	7	2.6%
Lin 2009 [55]	phase 2	242	Lapatinib	Single arm	1–2.56%; 3–4.43%; ≥5.11%	95%			2.4 (95% CI 1.9–3.3)	6.4 (95% CI 5.5–8.3)	6.2%
Wang 2021 [56]	retrospective	42	Lapatinib	Single arm	1.17.4%; 2.53.9%; 3.20.1%; ≥4.7.8%	59%			6.3 (95% CI 5.1–7.5)		31.0%
Gavilá 2019 [57]	retrospective	38	Lapatinib + Trastuzumab	Single arm	3 (2–4)				3.8	15.2	
Boccardo 2008 [58]	open label, prospective	138	Lapatinib + Capecitabine	Single arm	≥2 100%						18.1%
Lin 2009 * [55]	phase 2 (expansion)	50	Lapatinib + Capecitabine	Single arm	2	95%			3.7 (95% CI 2.4–4.4)	NR	20.0%
Sutherland 2010 [59]	open label, prospective	34	Lapatinib + Capecitabine	Single arm	mean 2.4 (Range 1–5)	94%			5.1 (95% CI 3.5–6.5)	NR	20.6%
Metro 2011 [60]	retrospective	30	Lapatinib + Capecitabine	Single arm	median 2 (Range 1–5)	87%	97%	40% > 3	5.1 (95% CI 2.6–7.5)	11 (95% CI 4.3–17.6)	31.8%
Lin 2011 [61]	phase 2, randomised	13	Lapatinib + Capecitabine (13)	Lapatinib + Topotecan (9)	>1	100%	59%		NR	NR	38.5%
Cetin 2012 [62]	retrospective	85	Lapatinib + Capecitabine	Single arm	>3.74.1%	100%	96.5%		7.0 (95% CI 5.0–10.0)	13 (95% CI 9–17)	27.1%
Bachelot 2013-LANDSCAPE [63]	phase 2	44	Lapatinib + Capecitabine	Single arm	1–2.78%; 3–4.22%	0%	84%	median 3 (Range 1–25)	5.5 (95% CI 4.3–6.0)	17 (95% CI 13.7–24.9)	56.8%
Ro 2012 [64]	open label, prospective	58	Lapatinib + Capecitabine	Single arm	>3.38%	91%			4.5 (95% CI 4.2–5.5)	12.2 (9.9–14.5)	17.0%
Dubianski 2014 [65]	retrospective	19	Lapatinib + Capecitabine	Single arm					8.1		
Shawky 2014 [66]	phase 2	21	Lapatinib + Capecitabine	Single arm	>2.100%	76%	91%	57% > 3	5.5 (Range 1.1–22.0)	11	33.3%
Krop 2015-Emilia [45]	phase 3b (posthoc)	50	Lapatinib + Capecitabine (50)	Trastuzumab-emtansine (45)	median 3 (Range 1–13)	70%	79%		5.7	12.9	
Kaplan 2014 [67]	retrospective	46	Lapatinib + Capecitabine	Single arm	>2.48.9%	96%	86.5%	48% > 3		19.1	36.9%
Gui 2020 [68]	retrospective	14	Lapatinib + Capecitabine	Single arm	>3.82.6%	100%			8.4 (95% CI 2.2–14.7)		35.7%
Seligmann 2020-LANTERN [69]	phase 2, randomised	16	Lapatinib + Capecitabine (16)	Trastuzumab + Capecitabine (14)		100%	70%		6.2 (95% CI 3.6–7.1)	NR	25.0%
Hurvitz 2021-NALA [34]	phase 3b (posthoc)	50	Lapatinib + Capecitabine (50)	Neratinib + Capecitabine (51)	2.68%; ≥3.32%	80%	84%		4.3 (95% CI 2.8–5.6)	12.4 (95% CI 9.7–16.9)	28.2%
Yang 2021 [70]	retrospective	25	Lapatinib + Chemo (71%) Capecitabine)	Pyrotinib + Chemo (80% Capecitabine)					3.5		

* expansion cohort of Lin 2009.

**Table 4:** Characteristics of included Pyrotinib studies (n = 9).

Study	Phase	Patients with Baseline BM (*n*)	Intervention (*n*)	Control (*n*)	Line of Therapy	Previous Local Treatment for BM (%)	Extra CNS Disease (%)	Number of BM	mPFS (Months)	mOS (Months)	CNS ORR %
Yan 2020-Phenix [71]	phase 3	21	Pyrotinib + Capecitabine (21)	Capecitabine (10)					6.9 (95% CI 5.4–NR)		
Y.Lin 2020 [72]	retrospective	31	Pyrotinib + Capecitabine (59%)/Other * (41%)	Single arm	1–2.38% 3.22% ≥4.40%	55%	88,50%		6.7 (Range 4.7–8.7)		28.0%
Gao 2021 [73]	retrospective	42	Pyrotinib (+Other 59%)	Single arm	>1.93%	82%	90,00%	17% >5	11.1		47.6%
Zhang 2021 [74]	retrospective	21	Pyrotinib + Capecitabine (55%)/Other (38%)/Mono (7%)	Single arm	>1.88%				16.6 (95% CI 13.7–24.1)		45.5%, only 50% measurable disease
Yang 2021 [70]	retrospective	13	Pyrotinib + Other (80% Capecitabine) (13)	Lapatinib+ Chemo (71% Capecitabine) (35)					6.5		
Anwar 2021 [75]	retrospective	39 (2 cohorts combined)	Pyrotinib + Capecitabine (64%)/Other (36%)	Single arm	>3.62%	43% (of both cohorts)			8.7 (95% CI 6.4–11.9)	13.9	28.2% = 24% of 17.31% of 22
C.Li 2021 [76]	retrospective	53	Pyrotinib + Capecitabine (35%)/ Other (63%)/ Mono (3%)	Single arm		77%			7.0 (Range 6.1–7.8)		43.4%
Y.Li 2021 [77]	retrospective	23	Pyrotinib + Vinorelbine	Single arm					6.3 (Range 3.4–9.2)		
Yan 2022—Permeate [78]	phase 2	78	Pyrotinib + Capecitabine	Single arm		76%					66.7%

* other = other chemotherapy.

## Data Availability

Data collected for this study is readily available, as all included articles in this meta-analysis are publicly accessible through PubMed, EMBASE, Web of Science and The Cochrane Library.

## Supplementary Materials

The following supporting information can be downloaded at: https://www.mdpi.com/article/10.3390/cancers14225612/s1, Figure S1: Sensitivity analysis (excluding abstract-only articles) pooled ORR (%) meta-analysis; A. Trastuzumab-emtansine (T-DM1); B. Lapatinib + capecitabine (LC); Figure S2: Sensitivity analysis (excluding abstract-only articles) pooled mOS (months) meta-analysis: Lapatinib + capecitabine (LC); File S1: Table S1: Search strategy in PubMed; Table S2: Search strategy in Embase.com; Table S3: Search strategy in Clarivate Analytics/Web of Science Core Collection; Table S4: Search strategy in Wiley/Cochrane Library; File S2: PRISMA_2020_checklist.
