# Supplementary material for: Systemic Therapy for Patients with HER2-Positive Breast Cancer and Brain Metastases: A Systematic Review and Meta-Analysis
Source: Cancers (Basel). 2022 Nov 15;14(22):5612. doi: 10.3390/cancers14225612 (PMC9688214; doi:10.3390/cancers14225612)

Supplementary Figure S1: Sensitivity analysis (excluding abstract-only articles) pooled ORR (%) meta-analysis A. Trastuzumab-emtansine (T-DM1); B. Lapatinib + capecitabine (LC)

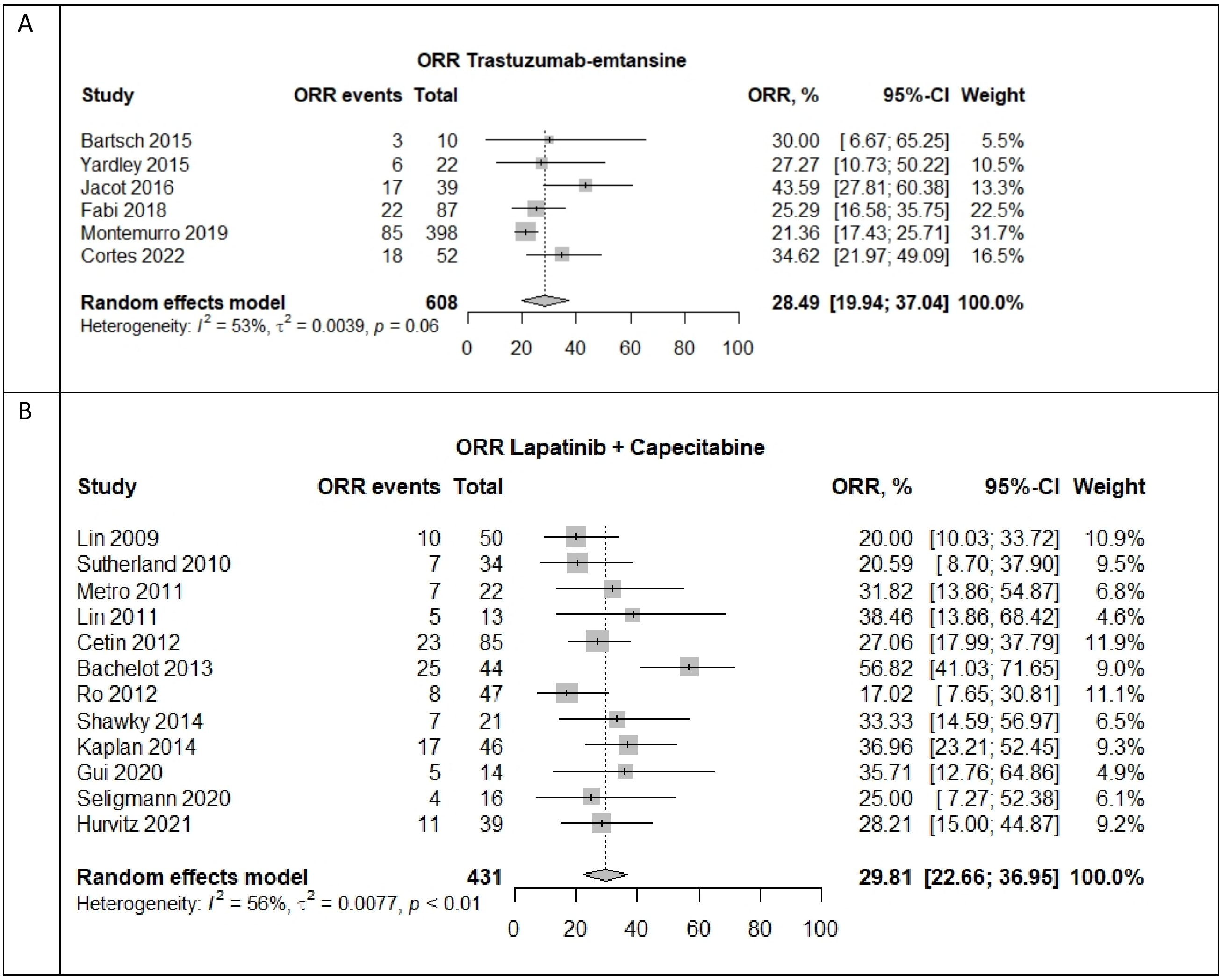

Supplement: Supplementary file 1 [file cancers-14-05612-s001.zip › Supplementary Figure S1. Sensitivity analysis pooled ORR.pdf]
