# Supplementary material for: Systemic Therapy for Patients with HER2-Positive Breast Cancer and Brain Metastases: A Systematic Review and Meta-Analysis
Source: Cancers (Basel). 2022 Nov 15;14(22):5612. doi: 10.3390/cancers14225612 (PMC9688214; doi:10.3390/cancers14225612)
Supplement: Supplementary file 1 [file cancers-14-05612-s001.zip › Supplementary File S1. Search strategy.pdf]

METHODS SECTION

A systematic search was performed in the databases: PubMed, Embase.com, Clarivate Analytics/Web of Science Core Collection and the Wiley/Cochrane Library. The timeframe within the databases was from inception to 19th January 2022 and conducted by GBL and IW. The search included keywords and free text terms for (synonyms of) 'breast neoplasm' combined with (synonyms of) ‘erythroblastic oncogene B’ combined with (synonyms of) ‘brain metastasis’. Reviews, animal studies, comments, letters, editorials, qualitative studies, case reports and case series were excluded from the search. A full overview of the search terms per database can be found in the supplementary information (see appendix 1). No limitations on date or language were applied in the search.

My Details:

My name: George Louis Burchell

ORCID: https://orcid.org/0000-0002-6281-4179

My affiliation: Medical Library, Amsterdam UMC / Vrije Universiteit, Location VUmc, De Boelelaan 1117, 1081 HV, Amsterdam, The Netherlands.

Supplementary File S1

Table S1: Search strategy in PubMed

Search	Query	Results
**#9**	#8 NOT ("Case Reports"[Publication Type] OR "case report*"[tiab] OR "case stud*"[tiab] OR "case histor*"[tiab] OR "case serie*"[tiab])	**556**
**#8**	#7 NOT ("Qualitative Research"[Mesh] OR "Focus Groups"[Mesh] OR "Interview" [Publication Type] OR "Interviews as Topic"[Mesh] OR "Narration"[Mesh] OR "Personal Narratives as Topic"[Mesh] OR "Observational Studies as Topic"[Mesh] OR "Observational Study"[Publication Type] OR "Tape Recording"[Mesh] OR "Grounded Theory"[Mesh] OR "thematic analys*"[tiab] OR "content analys*"[tiab] OR "focus group*"[tiab] OR "ethnograph*"[tiab] OR "ethnograf*"[tiab] OR "etnograf*"[tiab] OR "field stud*"[tiab] OR "phenomenolog*"[tiab] OR "narration*"[tiab] OR "narrative"[tiab] "case stud*"[tiab] OR "qualitative stud*"[tiab] OR "qualitative analys*"[tiab] OR "qualitative research*"[tiab] OR "qualitative method*"[tiab] OR "multimethodolog*"[tiab] OR "mixed method*"[tiab] OR "observation*"[tiab] OR "grounded theor*"[tiab] OR "audio recording*"[tiab] OR "tape recording*"[tiab] OR "audiotape*"[tiab] OR (("semi-structured"[tiab] OR "semistructured"[tiab] OR "unstructured"[tiab] OR "informal"[tiab] OR "in-depth"[tiab] OR "indepth"[tiab] OR "face-to-face"[tiab] OR "structured"[tiab] OR "guide*"[tiab]) AND ("interview*"[tiab] OR "discussion*"[tiab] OR "questionnaire*"[tiab])))	**629**
**#7**	#6 NOT ("Comment" [Publication Type] OR "Letter" [Publication Type] OR "Editorial" [Publication Type])	**650**
**#6**	#5 NOT ("Animals"[Mesh] NOT "Humans"[Mesh])	**683**
**#5**	#4 NOT ("systematic review"[tiab] OR "systematic literature review*"[tiab] OR "review*"[tiab] OR "Review"[Publication Type] OR "Meta-Analysis as Topic"[Mesh] OR "meta-analysis"[tiab] OR "Meta-Analysis"[Publication Type])	**698**
**#4**	#1 AND #2 AND #3	**976**

Search	Query	Results
**#3**	"central nervous system metasta*"[tiab] OR "CNS metasta*"[tiab] OR "brain metasta*"[tiab] OR "metastasis to the brain*"[tiab] OR "metastasized to the brain"[tiab] OR "metastasised to the brain"[tiab] OR "metastasis to the CNS*"[tiab] OR "metastasized to the CNS"[tiab] OR "metastasised to the CNS"[tiab] OR "metastasis to the central nervous system*"[tiab] OR "metastasized to the central nervous system*"[tiab] OR "metastasised to the central nervous system*"[tiab]	**16,228**
**#2**	"Genes, erbB-2"[Mesh] OR "ERBB2 protein, human"[Supplementary Concept] OR "erythroblastic oncogene B"[tiab] OR "erbB2*"[tiab] OR "erbB 2*"[tiab] OR "Erbb2"[tiab] OR "Erbb 2"[tiab] OR "neugene*"[tiab] OR "neu gene*"[tiab] OR "proto-oncogene Neu*"[tiab] OR "human epidermal growth factor receptor 2*"[tiab] OR "HER2*"[tiab] OR "HER 2*"[tiab] OR "HER2/neu"[tiab] OR "CD340"[tiab] OR "CD 340"[tiab]	**50,345**
**#1**	"Breast Neoplasms"[Mesh] OR (("breast*"[tiab] OR "mamma*"[tiab]) AND ("cancer*"[tiab] OR "carcinom*"[tiab] OR "malignan*"[tiab] OR "metasta*"[tiab] OR "neoplas*"[tiab] OR "tumor*"[tiab] OR "tumour*"[tiab]))	**504,112**

# = number of search step, used in Wiley/Cochrane Library, *is added standard in open text- search terms in Wiley/Cochrane Library to broaden the search.

Table S2: Search strategy in Embase.com

Search	Query	Results
**#9**	#8 NOT ('case report'/exp OR (“case report*” OR “case stud*” OR “case histor*” OR “case serie*”):ti,ab,kw)	**1,092**
**#8**	#7 NOT ('qualitative research'/exp OR 'interview'/exp OR 'narrative'/exp OR 'observational study'/exp OR 'recording'/exp OR 'grounded theory'/exp OR (‘thematic analys*’ OR ‘content analys*’ OR ‘focus group*’ OR ‘ethnograph*’ OR ‘ethnograf*’ OR ‘etnograf*’ OR ‘field stud*’ OR ‘phenomenolog*’ OR ‘narration*’ OR ‘narrative’ ‘case stud*’ OR ‘qualitative stud*’ OR ‘qualitative analys*’ OR ‘qualitative research*’ OR ‘qualitative method*’ OR ‘multimethodolog*’ OR ‘mixed method*’ OR ‘observation*’ OR ‘grounded theor*’ OR ‘audio recording*’ OR ‘tape recording*’ OR ‘audiotape*’):ti,ab,kw OR ((‘semi-structured’ OR ‘semistructured’ OR ‘unstructured’ OR ‘informal’ OR ‘in-depth’ OR ‘indepth’ OR ‘face-to-face’ OR ‘structured’ OR ‘guide*’):ti,ab,kw AND (‘interview*’ OR ‘discussion*’ OR ‘questionnaire*’):ti,ab,kw))	**1,248**
**#7**	#6 NOT ('conference abstract'/it OR 'conference review'/it OR 'editorial'/it OR 'erratum'/it OR 'letter'/it OR 'note'/it OR 'short survey'/it))	**1,304**
**#6**	#5 NOT ([animals]/lim NOT [humans]/lim)	**2,493**
**#5**	#4 NOT ('systematic review'/exp OR 'meta analysis'/exp OR (‘systematic literature review’ OR ‘systematic review*’ OR ‘meta-analys*’ OR ‘review*’):ab,ti,kw)	**2,605**
**#4**	#1 AND #2 AND #3	**3,352**
**#3**	'central nervous system metastasis'/exp OR (‘central nervous system metasta*’ OR ‘CNS metasta*’ OR ‘brain metasta*’ OR ‘metastasis to the brain*’ OR ‘metastasized to the brain’ OR ‘metastasised to the brain’ OR ‘metastasis to the CNS*’ OR ‘metastasized to the CNS’ OR ‘metastasised to the CNS’ OR ‘metastasis to the central nervous system*’ OR ‘metastasized to the central nervous system*’ OR ‘metastasised to the central nervous system*’):ti,ab,kw	**50,534**

Search	Query	Results
**#2**	'oncogene neu'/exp OR 'epidermal growth factor receptor 2'/exp OR 'human epidermal growth factor receptor 2 positive breast cancer'/exp OR (‘erythroblastic oncogene B’ OR ‘erbB2*’ OR ‘erbB 2*’ OR ‘Erbb2’ OR ‘Erbb 2’ OR ‘neugene*’ OR ‘neu gene*’ OR ‘proto-oncogene Neu*’ OR ‘human epidermal growth factor receptor 2*’ OR ‘HER2*’ OR ‘HER 2*’ OR ‘HER2/neu’ OR ‘CD340’ OR ‘CD 340’):ti,ab,kw	**108,102**
**#1**	'breast tumor'/de OR 'breast cancer'/exp OR 'experimental mammary neoplasm'/exp OR ((‘breast*’ OR ‘mamma*’):ti,ab,kw<br>AND (‘cancer*’ OR ‘carcinom*’ OR ‘malignan*’ OR ‘metasta*’ OR ‘neoplas*’ OR ‘tumor*’ OR ‘tumour*’):ti,ab,kw)	**760,299**

# = number of search step, used in Wiley/Cochrane Library, *is added standard in open text- search terms in Wiley/Cochrane Library to broaden the search.

Table S3: Search strategy in Clarivate Analytics/Web of Science Core Collection

Search	Query	Results
**#7**	#6 NOT TS=("case report*" OR "case stud*" OR "case histor*" OR "case serie*")	**1,031**
**#6**	#5 NOT TS=("thematic analys*" OR "content analys*" OR "focus group*" OR "ethnograph*" OR "ethnograf*" OR "etnograf*" OR "field stud*" OR "phenomenolog*" OR "narration*" OR "narrative" "case stud*" OR "qualitative stud*" OR "qualitative analys*" OR "qualitative research*" OR "qualitative method*" OR "multimethodolog*" OR "mixed method*" OR "observation*" OR "grounded theor*" OR "audio recording*" OR "tape recording*" OR "audiotape*" OR (("semi-structured" OR "semistructured" OR "unstructured" OR "informal" OR "in-depth" OR "indepth" OR "face-to-face" OR "structured" OR "guide*") AND ("interview*" OR "discussion*" OR "questionnaire*")))	**1,063**
**#5**	#4 NOT TS=("systematic review" OR "systematic literature review*" OR "review*" OR "meta-analysis")	**1,090**
**#4**	#1 AND #2 AND #3	**1,379**
**#3**	TS=("central nervous system metasta*" OR "CNS metasta*" OR "brain metasta*" OR "metastasis to the brain*" OR "metastasized to the brain" OR "metastasised to the brain" OR "metastasis to the CNS*" OR "metastasized to the CNS" OR "metastasised to the CNS" OR "metastasis to the central nervous system*" OR "metastasized to the central nervous system*" OR "metastasised to the central nervous system*")	**22,331**
**#2**	TS=("erythroblastic oncogene B" OR "erbB2*" OR "erbB 2*" OR "Erbb2" OR "Erbb 2" OR "neugene*" OR "neu gene*" OR "proto-oncogene Neu*" OR "human epidermal growth factor receptor 2*" OR "HER2*" OR "HER 2*" OR "HER2/neu" OR "CD340" OR "CD 340")	**64,660**

Search	Query	Results
**#1**	TS=(((“breast*” OR “mamma*”) AND (“cancer*” OR “carcinom*” OR “malignan*” OR “metasta*” OR “neoplas*” OR “tumor*” OR “tumour*”)))	**725,440**

# = number of search step, used in Wiley/Cochrane Library, *is added standard in open text- search terms in Wiley/Cochrane Library to broaden the search.

Table S4: Search strategy in Wiley/Cochrane Library

Search	Query	Results
**#4**	#1 AND #2 AND #3	**1**
**#3**	(“central nervous system metasta*” OR “CNS metasta*” OR “brain metasta*” OR “metastasis to the brain*” OR “metastasized to the brain” OR “metastasised to the brain” OR “metastasis to the CNS*” OR “metastasized to the CNS” OR “metastasised to the CNS” OR “metastasis to the central nervous system*” OR “metastasized to the central nervous system*” OR “metastasised to the central nervous system*”):ti,ab,kw	**14**
**#2**	(“erythroblastic oncogene B” OR “erbB2*” OR “erbB 2*” OR “Erbb2” OR “Erbb 2” OR “neugene*” OR “neu gene*” OR “proto-oncogene Neu*” OR “human epidermal growth factor receptor 2*” OR “HER2*” OR “HER 2*” OR “HER2/neu” OR “CD340” OR “CD 340”):ti,ab,kw	**7,240**
**#1**	((“breast*” OR “mamma*”):ti,ab,kw AND (“cancer*” OR “carcinom*” OR “malignan*” OR “metasta*” OR “neoplas*” OR “tumor*” OR “tumour*”):ti,ab,kw)	**39,812**

# = number of search step, used in Wiley/Cochrane Library, *is added standard in open text- search terms in Wiley/Cochrane Library to broaden the search.
